# Characterization of secondary care for COPD in Sweden

**DOI:** 10.1080/20018525.2016.1270079

**Published:** 2017-01-24

**Authors:** Josefin Sundh, Christer Janson, Gunnar Johansson, Anders Lindén, Claes-Göran Löfdahl, Thomas Sandström, Kjell Larsson

**Affiliations:** ^a^ Department of Respiratory Medicine, School of Medical Sciences, Örebro University, Örebro, Sweden; ^b^ Department of Medical Sciences: Respiratory; Allergy and Sleep Research, Uppsala University, Uppsala, Sweden; ^c^ Department of Public Health and Caring Science, Family Medicine and Preventive Medicine, Uppsala University, Uppsala, Sweden; ^d^ Unit for Lung and Airway Research, Institute of Environmental Medicine, Karolinska Institutet, Stockholm, Sweden; ^e^ Department of Respiratory Medicine and Allergology, Lund University, Lund, Sweden; ^f^ Department of Public Health and Clinical Medicine, Division of Medicine, Umeå University, Umeå, Sweden

**Keywords:** GOLD classification, pharmacological therapy, structural resources, patient education

## Abstract

**Introduction:** Only a selected proportion of chronic obstructive pulmonary disease (COPD) patients are managed in secondary care. The aim of this study was to characterize disease severity, treatment and structure of secondary care for COPD in Sweden.

**Methods:** Information was collected from 29 of 33 existing secondary care units of respiratory medicine in Sweden, using both individual data from 373 consecutively enrolled COPD patients with Global initiative on Obstructive Lung Disease (GOLD) stage III–IV and a structural questionnaire about available resources at the units. Patient data included exacerbations, health status assessed by COPD Assessment Test (CAT), lung function, comorbid conditions, pharmacological treatment and vaccinations. Structural data included available smoking cessation support, multidisciplinary rehabilitation, physical training, patient education and routine follow-up after exacerbations at the respective unit. All patients were reclassified according to the GOLD 2014 group A–D classification. Multiple linear regression investigated associations of available resources with number of exacerbations and CAT score.

**Results:** According to GOLD 2014, 87% of the population were GOLD D and 13% were GOLD C. Triple inhaled therapy were prescribed in 88% of the patients. Over 75% of the units had resources for smoking cessation, multidisciplinary rehabilitation, physical training and patient education. Routine follow-up after exacerbations was available in 35% of the units. Being managed at units with access to structured patient education was associated with statistically significantly fewer exacerbations (adjusted regression coefficient (95% confidence interval) −0.79 (−1.39 to −0.19), *p* = 0.010).

**Conclusion**: Most stage III–IV COPD patients managed at secondary care respiratory units in Sweden have maximized inhaled therapy and high risk disease even when reclassified according to GOLD 2014. Most units have access to smoking cessation, rehabilitation and patient education. Patients managed at units with structured patient education have a lower exacerbation risk.

## Introduction

The prevalence of chronic obstructive pulmonary disease (COPD) in the adult population in Sweden is approximately 10%,[1–3] which is in accordance with global reports.[4] The majority of Swedish COPD patients are handled in primary care, but as previously reported the most severe and resource utilizing patients are found in secondary care.[5] However, the management of COPD in secondary care has previously not been surveyed in detail.

The major components of COPD management are smoking cessation, pharmacological treatment and pulmonary rehabilitation. All COPD patients should be correctly classified for disease severity and future risk of exacerbations, and receive optimized therapy according to current recommendations. Previously, Swedish national recommendations of treatment were based on the spirometric classification stage I to IV from Global initiative on Obstructive Lung Disease (GOLD) 2011, but since 2015 the updated guidelines of the National Board of Health and Welfare [6] and the Medical Product Agency [7] recommend a treatment schedule based on the recent GOLD COPD risk evaluation groups A–D, including lung function, symptoms and exacerbation frequency.[8] It is also of utter importance that COPD care can offer support for smoking cessation, multidisciplinary rehabilitation including physical training, structured patient education and follow-up, in particular after exacerbations.[6–8]

The aim of the current study was to characterize the disease severity, treatment and structure of secondary respiratory care for COPD in Sweden.

## Methods

### Data collection

In Sweden, there are 33 hospital-based secondary care units for respiratory medicine, including departments of respiratory medicine or sections of respiratory medicine in departments of internal medicine. All 33 units were invited to participate in the present study. Data collection included both information from a structural questionnaire to the physician responsible for COPD care, and information from patient visits. Each respiratory unit was asked to consecutively enroll a maximum of 10 patients with GOLD grade III COPD and five patients with GOLD grade IV COPD [8] during the period from 12 May 2011 to 28 March 2012. The proportions of stage III and IV patients were chosen to approximately match the distribution of severe and very severe COPD as assessed in the general population.[9,10] During the patients’ visits, information was collected by the physician on patient demographics, treatment, comorbidities, exacerbations, and health status. The information was entered into the study case record form together with data from the most recently performed spirometry. Post-bronchodilator values were used, but replaced with pre-bronchodilator values if post-bronchodilator values were missing (43%). The only exclusion criterion was an inability to complete the study on language grounds. The analyses of health status, comorbidities and frequent exacerbations have been described and published elsewhere.[11,12]

### Variables

Patient data used in this study included sex, age, smoking history and habits, forced expiratory volume in one second in percentage of predicted value (FEV_1_% pred), body weight and height, number of exacerbations including hospitalizations due to exacerbations, health status using COPD assessment test (CAT), comorbidity, current pharmacological treatment and influenza and pneumococcal vaccination status.

An exacerbation was defined as worsening of symptoms of dyspnea and sputa beyond normal day to day variation.[13] Frequent exacerbations were defined as having two or more exacerbations or one or more hospitalizations due to COPD exacerbations in the previous 12 months.[8] The patients were classified into the following three groups according to BMI: BMI < 22, 22 ≤ BMI < 30 and BMI ≥ 30. The cut off-values were based on the prognostic importance of BMI in COPD reported in previous studies.[14] Symptoms were categorized as less (CAT < 10) or more (CAT ≥ 10).[8] Comorbid conditions were defined as conditions requiring medical treatment, and included cardiovascular disease, diabetes, renal dysfunction, musculoskeletal symptoms, osteoporosis and depression. COPD treatment groups included: long-acting muscarinic antagonists, long-acting beta-2-agonists, combined bronchodilators, combined long-acting beta-2-agonists and inhaled corticosteroids, triple inhaled therapy with long-acting muscarinic antagonists, long-acting beta-2-agonists and inhaled corticosteroids, long term oxygen therapy (LTOT) and roflumilast. Influenza vaccination referred to the previous year and pneumococcal vaccination to the previous five years.

Data from the structural questionnaires included information on the specific COPD guidelines that were used for disease management, and on access to smoking cessation support, multidisciplinary rehabilitation, physical training, patient education and planned follow-up after exacerbations, at the respective respiratory unit.

### Statistics

Statistical analyses were performed using IBM Statistics SPSS version 22.0 (SPSS Inc., Chicago, IL, USA). The proportion of patients with different characteristics and pharmacological treatments were calculated. All patients were classified as GOLD 2011 stage III or IV, and reclassified as GOLD groups C or D according to GOLD 2014. Cross-tabulations and chi-squared test explored the association between patient characteristics with group C or D, and treatments with group C or D. Proportion of participating units with available COPD education, multidisciplinary rehabilitation, physical training, smoking cessation and planned follow-up at the own unit after exacerbations were calculated, as well as percentages of units using different treatment recommendations. Associations between available resources and total number of exacerbations and CAT score were analyzed by means of linear regression. The multiple model included sex, age and patients’ characteristics with statistically significant associations in the univariate model. In all analyses, a *p*-value below 0.05 was considered as statistically significant.

### Ethics

The study was conducted as a non-interventional trial, in accordance with EU directive 2001/20/EC and the Declaration of Helsinki. The study protocol and the study was reviewed and approved by the Regional Ethical Review Board of Umeå University (Dnr 2011-10-31M). Oral and written informed consent was given by all patients.

## Results

### Classification of disease severity and patient characteristics

**Figure 1.**
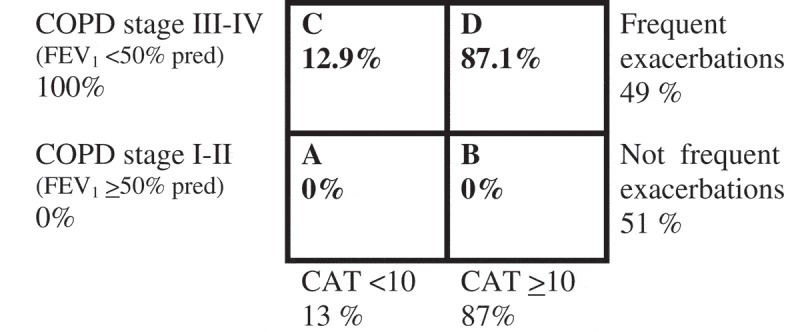
Reclassification of severity for the patient population according to GOLD 2014 risk evaluation groups A–D.

As only patients with COPD stage III–IV were included, 100% of the patients fulfilled the spirometric criterion for a high risk according to the present GOLD classification and were classified as either group C or D. Only 13% had minor symptoms (CAT < 10), and subsequently 87% of the population were classified as group D. In addition, 49% of the patients had frequent exacerbations (Figure 1).

**Figure 2.**
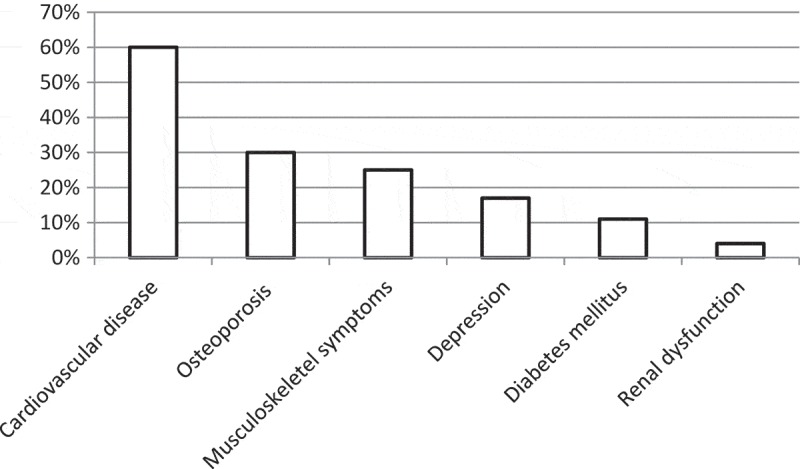
Comorbid conditions. Frequency of comorbid conditions in the patient population.

**Figure 3.**
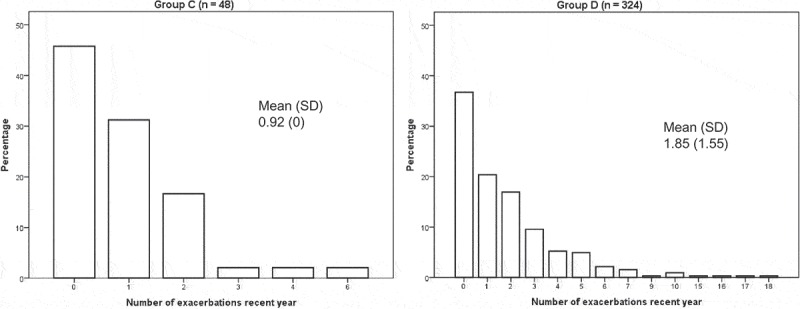
Exacerbation frequency in GOLD 2014 groups. Percentage of patients distributed over number of exacerbations during the previous year. SD = standard deviation.

There were no differences in sex, age, BMI, comorbidities or smoking status, although the accumulated exposure to tobacco and exacerbations frequency in the previous year was statistically significantly higher in group D (Table 1). In the total population, the prevalence of current smoking was 16%, and the number of comorbid conditions varied from 1 to 4. The most common comorbidity was cardiovascular disease (Figure 2). The mean exacerbation frequency in the previous year was 1.7 (SD 2.4), but varied from 0 to 18 (Figure 3).

**Table 1.** Patient characteristics.

	**COPD group C** n = 48 (12.8%)	**COPD group D** n = 325 (87.1%)	*p*
Female sex	23 (47.9%)	185 (56.9%)	0.24
Age	72.0 (8.19)	71.1 (7.80)	0.21
Current smoking	8 (16.7%)	53 (16.3%)	0.95
Cumulative exposure (pack years)	33.7 (28.7)	34.3 (16.7)	0.03
BMI			
<22	15 (31.3%)	99 (30.5%)	0.60
≥22, <30	29 (60.4%)	182 (56.0%)	
≥30	4 (8.3%)	44 (13.5%)	
Number of exacerbations in the previous year	0.9 (1.2)	1.9 (2.6)	0.002
Cardiovascular disease	30 (62.5%)	193 (59.9%)	0.74
Diabetes	3 (6.3%)	37 (11.5%)	0.28
Renal impairment	2 (4.2%)	12 (3.8%)	0.89
Musculoskeletal disease	9 (18.8%)	81 (25.5%)	0.31
Osteoporosis	9 (19.1%)	94 (31.3%)	0.09
Depression	4 (8.3%)	58 (81.1%)	0.09

Patient characteristics distributed over severity group (GOLD 2014).

Data presented as number (%) or mean (standard deviation).

### Pharmacological treatment and vaccinations

**Figure 4.**
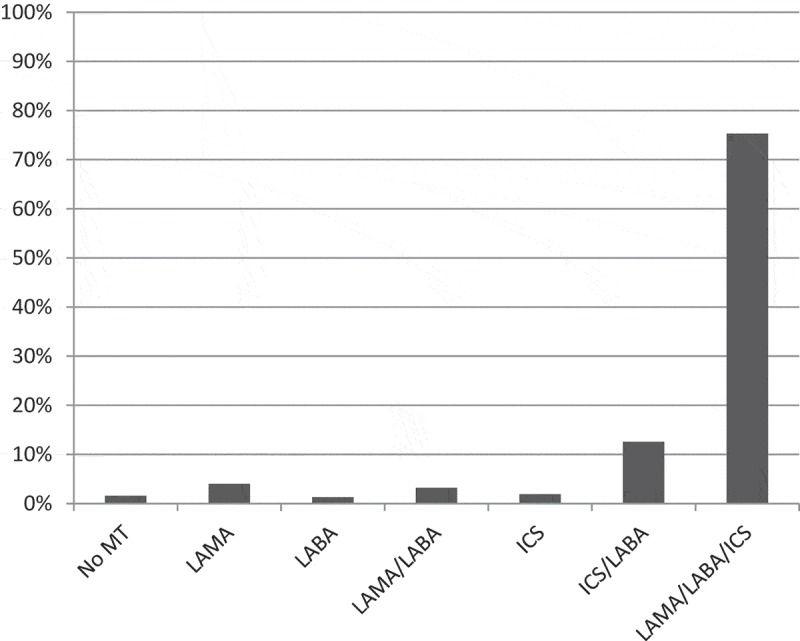
Inhalation therapy. Inhalation therapy groups distributed over the patient population. No MT = no maintenance therapy, LAMA = long-acting muscarinic antagonists, LABA = long-acting beta-2-agonists, ICS = inhaled corticosteroids.

The most common maintenance treatment was triple inhaled therapy with long-acting muscarinic antagonists, long-acting beta-2-agonists and inhaled corticosteroids present in 75.3% of the patients (Figure 4). Maintenance treatment with only bronchodilators was more common in stage III compared to stage IV, and in group C compared with group D. Maintenance treatment with inhaled corticosteroids with or without bronchodilators was more common in stage IV than in stage III, and in group D than in group C (Table 2). Add-on treatment with LTOT was present in 46 patients (12.3%). Roflumilast was prescribed in 27 patients (7.2%) and 30% of these (*n* = 8) fulfilled the current indications for roflumilast in Sweden (FEV_1_% pred < 50, frequent exacerbations and chronic bronchitis). As for vaccinations, 75.6% had received influenza vaccination in the previous year and 63% had received pneumococcal vaccination in the previous five years. Influenza and pneumococcal vaccination was more common in group D than C but did not differ between stage III and IV (Table 2).

**Table 2.** Pharmacological interventions by disease severity.

	III *n* = 259 (69.4%)	IV *n* = 114 (30.6%)	*p*	C *n* = 48 (12.9%)	D *n* = 325 (87.1%)	*p*
Only bronchodilators (LAMA and/or LABA)	29 (11.2%)	3 (2.6%)	0.007	9 (18.8%)	23 (7.1%)	0.007
Inhaled corticosteroids (ICS) with or without bronchodilators	225 (86.9%)	110 (96.5%)	0.005	39 (81.3%)	296 (91.1%)	0.036
Triple inhaled therapy (LAMA, LABA and ICS)	196 (75.7%)	85 (74.6%)	0.818	31 (64.6%)	250 (76.9%)	0.064
Influenza vaccination	198 (76.4%)	84 (73.7%)	0.567	30 (62.5%)	252 (77.5%)	0.024
Pneumococcal vaccination	156 (60.2%)	79 (69.3%)	0.095	24 (50%)	211 (64.9%)	0.046

Pharmacological treatment groups and vaccinations distributed over disease severity according to GOLD 2011 stage III/IV and GOLD 2014 group C/D. LAMA = long-acting muscarinic antagonists, LABA = long-acting beta-2-agonists, ICS = inhaled corticosteroids.

### Structure and resources in COPD care

According to the structural questionnaires, national guidelines from The National Board of Health and Welfare, the Swedish Medical Products Agency and the Swedish Respiratory Society were most commonly used, in 97% of the respiratory units. Local or regional guidelines were less common (28 and 14%, respectively). The international GOLD document was reported as a base for management in 45% of the units.

**Figure 5.**
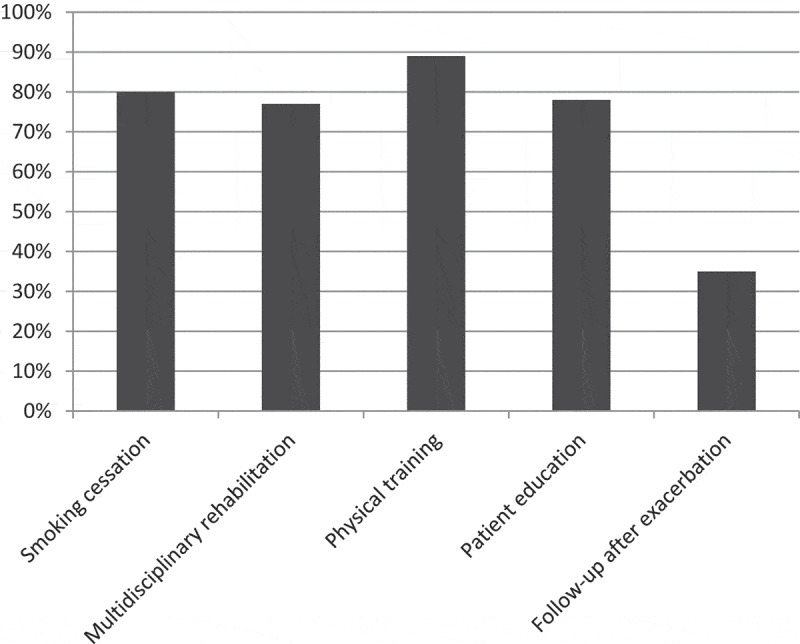
Resources at respiratory units. Proportions of secondary care units with available resources at the unit.

Smoking cessation, multidisciplinary rehabilitation, physical training and patient education were all available at more than 75% of the units. Routine follow-up visits after exacerbations at the respective secondary respiratory units were available in 35% of the participating units (Figure 5). In multiple linear regression analyses, patients managed at units with access to structured patient education had statistically significant exacerbations in the previous year (Table 3). No other significant association between access to resources and patient related outcomes such as exacerbations or health status were found (data not shown).

**Table 3.** Patient education and exacerbation frequency.

	Univariate linear regression (95% CI)	*p*	Multivariate linear regression (95% CI)	*p*
Female sex	0.37 (−0.13 to 0.87)	0.143	0.19 (−0.34 to 0.72)	0.486
Age	−0.004 (−0.04 to 0.03)	0.817	−0.02 (−0.06 to 0.01)	0.167
FEV_1_% pred	−0.04 (−0.07 to −0.01)	0.008	−0.02 (−0.05 to 0.02)	0.321
Comorbidity score	0.50 (0.31 to 0.69)	<0.0001	0.51 (0.31 to 0.70)	<0.0001
Patient education	−0.69 (−1.26 to −0.12)	0.017	−0.79 (−1.39 to −0.19)	0.010

The multivariate model included sex and age (a priori confounding factors) and comorbidity index and FEV_1_% pred (statistically significant in univariate analyses). FEV_1_% pred = forced expiratory volume in one second in percentage of predicted value. CI = confidence interval.

## Discussion

The first finding of this study is that stage III and IV COPD patients in Swedish secondary care often also suffer from frequent exacerbations and symptoms. By the time of the data collection the Swedish national guidelines of severity classification and treatment recommendations were based only on GOLD 2011 stages I–IV according to lung function measurement, and the present study included patients with stage III and IV. When the patients were reclassified according to GOLD 2014 risk staging, 87% had a severe disease with high risk and symptom burden (group D).

Secondly, most but not all patients had pharmacological treatment with both long-acting muscarinic antagonists, long-acting beta-2-agonists and inhaled corticosteroids, with no difference between group C and D. Treatment with only bronchodilators was more common in group C or lung function stage III, and treatment with inhaled corticosteroids was more common in group D and stage IV. Long-acting muscarinic antagonists [15] as well as inhaled corticosteroids in combination with long-acting beta-2-agonists have a well-documented effect on exacerbations.[16] More recent data have suggested a similar, or even better effect on exacerbations by double bronchodilation as from combined long-acting beta-2-agonists and inhaled corticosteroids.[17] However, we find it reasonable that the small but severely ill group of COPD patients managed in secondary care had optimized treatment with triple inhaled therapy, as this combination has been shown to have an addition effect on exacerbation frequency and symptoms in COPD.[18,19] Roflumilast had a limiting label and subsidization in Sweden at the time of data collection, which may explain its use in only 7% of the patients. Some 22% of the study patients (*n* = 82) had chronic bronchitis and frequent exacerbations which potentially could have indicated treatment with roflumilast. Among the few patients who were actually prescribed roflumilast, only a third had the documented phenotype and were prescribed roflumilast on an evidence based ground. We believe that the reason for this discrepancy is that physicians not always follow guidelines.

As for pharmacologic prevention with vaccinations, the coverage of influenza vaccination was higher than for pneumococcal vaccination. Influenza vaccination is administrated for free in Sweden, which could explain the difference from pneumococcal vaccination. In our opinion, the difference could also be justified by the much more solid scientific documentation for influenza vaccination than for pneumococcal vaccination in COPD.[20]

The third finding of interest is that access to smoking cessation, multidisciplinary rehabilitation, physical training and education is common, but still not available in all secondary care centers. Access to smoking cessation support, multidisciplinary rehabilitation, physical training and patient education was reported by more than 75% of the units whereas follow-up after exacerbations was only reported by 35% of the units. In comparison, the National COPD Resources and Outcomes Project in UK, including 100 larger sized hospitals, reported access to pulmonary rehabilitation in 89%, education programs in 89%, but full multidisciplinary rehabilitation in only 44%.[21] Physical training was the most common of the investigated resources in Swedish secondary care respiratory units. This is encouraging, as physical training has been shown to improve dyspnea, health related quality of life, physical capacity [22] and, in association to exacerbations, even hospitalization and mortality.[23] Nevertheless, in our opinion physical training should be generally available at secondary care respiratory units for COPD.

Interestingly, access to patient education was statistically significantly associated with a reduced risk for exacerbations. Patient education can be applied in different ways; by lectures for groups of patients or by individual structured discussions.[24,25] One of the major aims with patient education is self-management; disease control through behavior change.[26] We speculate that the association of patient education with lower exacerbation frequency in our study population could be due to achievement of self-management skills. However, development of patient education in a center may also be a general marker of better and more intense management and treatment in all aspects. The finding is consistent with previous studies where a structured education system with the aim of increased self-management was associated with higher HRQL [27] and a reduced risk for hospitalized exacerbations.[28] Exacerbations of COPD are associated with increased decline in lung function and increased mortality, and thus follow-up after exacerbation is also crucial. The fact that a routine with follow-up at respective unit was present in only 35% and that the presence of follow-up was not associated with reduced exacerbation risk, may be related to a well-functioning system with follow-up by asthma/COPD nurses in Swedish primary care. Several Swedish studies have shown that access to or planned follow-up by a specific asthma COPD nurse in primary care is associated with reduced risk for exacerbations.[29–31]

Less than half of the secondary care respiratory units claimed to follow the international GOLD recommendations. In a survey of guideline-based COPD secondary care in Germany, a similar trend with greater adherence to national than international guidelines was observed.[32] We speculate that the preference of national guidelines in our study was due to the fact that during the time of data collection; national treatment recommendations were still based on the previous GOLD 2011 lung function stage I–IV classification, and did not incorporate the new ABCD classification. However, in reality not only lung function but also symptoms and exacerbations were influencing treatment choice.

### Strengths and limitations

Strengths of the current study include that it is a multicenter study with patients from almost all respiratory units in Sweden, and that it includes both patient related and structural data. Weaknesses include the fact that treatment information included only pharmacological treatment but not data on whether the individual patients actually received pulmonary rehabilitation or smoking cessation support even if available at the respective units.

## Conclusion

We conclude that most GOLD 2011 stage III–IV patients managed at secondary care units for COPD in Sweden have maximized inhaled therapy and high risk disease, even when reclassified to GOLD 2014 A–D groups. Many units have access to smoking cessation, rehabilitation and patient education. Patients managed at units with structured patient education have a lower exacerbation risk.
